# Lack of oral health awareness and interdisciplinary dental care: a survey in patients prior to endoprosthesis and orthopaedic centres in Germany

**DOI:** 10.1186/s12903-023-02793-7

**Published:** 2023-02-13

**Authors:** Gerhard Schmalz, Carina Lenzen, Florentine Reuschel, Fabian Fenske, Rainer Haak, Szymon Goralski, Andreas Roth, Dirk Ziebolz

**Affiliations:** 1 Department of Cariology, Endodontology and Periodontology, University Medical Center Leipzig, University of Leipzig, Liebigstr. 10-14, 04103 Leipzig, Germany; 2 Department of Craniomaxillofacial Surgery, University of Leipzig, Leipzig, Germany; 3 Department of Orthopaedics, Trauma and Plastic Surgery, University Hospital Leipzig, Leipzig, Germany

**Keywords:** Oral health behaviour, Endoprosthesis, Oral hygiene, Interdisciplinary collaboration

## Abstract

**Objectives:**

This cross-sectional survey aimed to evaluate the oral health behaviour of patients prior to endoprosthesis (EP), as well as the handling of oral health topics by German orthopaedic surgeons.

**Materials and methods:**

Consecutive patients prior to EP answered a questionnaire regarding oral health behaviour, oral hygiene, oral complaints and information on the relationship between EP and oral health. Another questionnaire was digitally mailed to orthopaedic centres throughout Germany. This questionnaire included the importance of oral health for EP and issues on dental referrals/consultations prior to EP.

**Results:**

A total of 172 patients were included in the study, of whom 35.5% of patients reported that they were informed about oral health and EP. Half of the individuals reported regular professional tooth cleaning, and less than one-third (29.1%) reported of the performance of interdental cleaning. Information on oral health and EP was associated with regular professional tooth cleaning (yes: 59.8% vs. no: 35.6%, *p* = 0.01). A total of 221 orthopaedic clinics were included in the study, of which only a few had dental contact (14%), although the majority (92.8%) of the clinics were familiar with causal relationships between oral health and EP infections. Less than half of the centres reported of either verbal (48%) or written (43.9%) referrals for their patients to the dentist. University Medical Centres reported of more frequent dental contacts (*p* < 0.01).

**Conclusion:**

Prior to EP, patients exhibited deficits in oral health behaviour, and orthopaedic clinics exhibited a lack of dental collaboration. Improvements in interdisciplinary care, especially regarding practical concepts for patient referral and education on oral health, appear to be necessary.

## Introduction

Perioprosthetic infections are very rare; however, due to the resulting high morbidity rate, endoprosthesis (EP) complications require very complex therapy by an interdisciplinary team, and they often have an unsatisfying prognosis [1]. Therefore, the prevention of such infections is a mandatory aim in pre- and postoperative care. As potential sources of infection, various bacterial foci may be able to cause haematogenous spread, thus leading to EP infection [2]. As one of these outcomes, the oral cavity and related inflammatory diseases have been repeatedly mentioned as being causes of EP infections; however, results of studies regarding this scenario have been controversial [3–5].

Although clear evidence for oral disease-induced EP infections remains unclear, several implications for dental care in patients prior to EP have been discussed. For example, dental clearance prior to EP surgery, such as the rehabilitation or removal of all potential infectious foci in the mouth, teeth, periodontium and jaws, has been mentioned as being highly relevant [6, 7]. Moreover, appropriate dental maintenance care, including prevention-oriented therapy via regular dental consultations and preventive measures to reduce the risk for de novo development of potential oral foci, can be regarded as a major task to reduce the risk of EP infections [6, 7]. In this context, the potential usage of antibiotic prophylaxis for dental interventions is controversial; additionally, recent literature clearly does not recommend antibiotic prophylaxis for patients with EP prior to dental measures [7, 8]. However, antibiotic prophylaxis may be reasonable in patients who are selected as being at-risk for EP infection [6]. Accordingly, uniform guidelines are still missing. Furthermore, regardless of the existing controversies, all of the abovementioned considerations require the following two circumstances: (I) patients need to be informed, should perform oral hygiene as appropriately as possible and should visit the dentist in a control/prevention-oriented manner; and (II) EP centres and dentists should work together and communicate to ensure appropriate dental care of the patients. For this scenario, referral of the patients for dental consultation should be applied, which has been previously reported as a concept [9]. Until now, these two issues have remained unclear. In particular, there is no information on whether the EP centres are aware of oral health concerns and whether they cooperate with dentists.

Therefore, the current questionnaire-based study had two aims. First, oral behaviour, oral hygiene, oral complaints and information on oral health (as well as its possible influence on EP) should be examined in patients prior to EP in one orthopaedic (endoprosthetic) centre in Germany. Second, German orthopaedic surgeons should be evaluated regarding their handling of oral health topics, potential patient referrals and available concepts to ensure appropriate dental care for the patients. To investigate these aims, two surveys were administered to patients and orthopaedic surgeons. It was hypothesised that patients are not aware of the potential importance of oral health for their EP; thus, they will not exhibit increased oral health behaviour. The second hypothesis was that EP centres rarely refer patients to a dentist and do not have a dental care concept for the respective patients prior to EP.

## Materials and methods

### Study design

This questionnaire-based cross-sectional study of patients prior to EP implantation and orthopaedic surgeons in Germany was performed in full accordance with the Declaration of Helsinki and was approved by the local ethics committee of Leipzig University (No: 116/20-ek). All of the participating patients provided their written informed consent. Moreover, orthopaedic surgeons who anonymously answered the questionnaire provided their informed consent for participation in the study.

### Patients

Within an interdisciplinary, cooperative project between the Department of Cariology, Endodontology and Periodontology and the Department of Orthopaedics, Trauma and Plastic Surgery, University Hospital Leipzig, Germany, patients were referred between April 2020 and December 2021 for dental examinations prior to EP surgery. Therefore, a risk classification concept was applied as previously described [9]. After informed consent was obtained, patients were asked to answer a series/number of questions within a composed questionnaire (see below). The inclusion criteria were age > 18 years, status prior to EP surgery and voluntary participation. The following exclusion criteria were utilized for the current study:

- Cognitive impairment, which would not allow for the answering of questions (e.g., severe dementia)
- Insufficient understanding of the German language, which would impair understanding of the study questions
- Previous EP implantations

Only questionnaires that were completely answered by the respective patients were included in the analysis.

### Orthopaedic centres

For this study, a cross-section of German orthopaedic centres that perform EP surgery were evaluated. The study aimed to include centres from all German districts to obtain a sample that was as representative as possible. To accomplish this, surgeons who were listed in the German Society for Orthopaedics and Trauma (DGOU) (n = 8000) were contacted and asked for their participation in the study. Only completed questionnaires were included in the analysis.

### Questionnaires

#### Patients

For the included patients, a questionnaire (in German) was compiled on oral health behaviour in general and in relation to EP. Therefore, a validated questionnaire from previous studies with other patient groups was used and adapted regarding oral health questions in the context of EP [10, 11]. The questionnaire included questions regarding oral health and oral hygiene behaviour, as well as the importance of oral health for EP, information on EP and oral health and oral complaints. Additionally, a medical history form assessed the age, sex, smoking status and medication intake of the included patients.

#### Orthopaedic centres

The questionnaire for the orthopaedic centres was composed based on similar previous studies [12, 13]. Therefore, the questionnaire content was adapted according to the context of EP with support and review by two experts from the German Society for Orthopaedics and Trauma (Deutsche Gesellschaft für Orthopädie und Unfallchirurgie e.V.: DGOU). Within the survey, information on the characteristics of the centres, the handling of oral health issues prior to and after EP surgery, the perceived importance for oral health in the context of EP and the need for antibiotic prophylaxis were assessed. The questionnaire was answered by 10 orthopaedic surgeons for validation and subsequently adapted according to the surgeons’ comments. The questionnaire was digitally performed by using the non-commercial program SoSci Survey (www.soscisurvey.de, SoSci Survey GmbH, Munich Germany). A link to the questionnaires was sent out in digital form by the office of the German Society for Orthopaedics and Trauma (DGOU) in December 2021 via e-mail to surgeons/members who were listed as members of the society. A second email was sent to each centre that had received the questionnaire and that had not responded within two weeks.

### Statistical analysis

Statistical analysis was performed with SPSS for Windows, Version 24.0 (SPSS Inc., US). Metric variables did not show a normal distribution, according to the Kolmogorov‒Smirnov test (*p* < 0.05). To compare two independent, nonnormally distributed samples, the Kruskal‒Wallis test was applied. Categorical and nominal data were analysed by using chi-square and Fisher’s tests, respectively. The significance level was set at *p* < 0.05.

## Results

### Patient’s perspective

A total of 172 patients were included in the current study, with a mean age of 66.58 ± 11.08 years; additionally, 50.6% of the patients were male, and 24.4% of the patients were current smokers. In the regular medical treatment of existing general diseases were 70.9% of the participants and 80.8% took medication regularly.

Less than 20% of the patients had oral complaints. Only a quarter of the patients stated that their dentist was informed about the planned EP; moreover, 35.5% of the participants were informed about oral health and EP, but 62.8% of the patients felt well educated. Only approximately half of the individuals reported regular professional tooth cleaning, and less than one-third (29.1%) of the patients stated that they performed interdental cleaning (Table 1).

**Table 1:** Results of the oral health questionnaire in patients before endoprosthesis (EP)

Parameter	Patients before EP (n = 172)
*Self-reported oral symptoms*	
Sensitive teeth	12.8
Bleeding gums	12.2
Bad taste	18.0
*Dental visiting and therapy before EP*	
Dental consultation prior to EP	18.6
Dentist knowledge on the planned EP	25.0
Regular professional tooth cleaning	48.3
Previous periodontal therapy	24.4
*Information about oral health and EP*	
Information on oral health and EP	35.5
Information from dentist	12.2
Information from orthopaedic surgeon	32.6
Information on oral hygiene and EP	31.5
Do you feel well educated on oral health?	62.8
*Personal oral behaviour/home care*	
Manual toothbrush	65.7
Powered toothbrush	35.5
Interdental cleaning	29.1
Oral rinse	40.7
Fluoride gel	3.5

All values are given as percentages (%)

### Associations between patient information and oral complaints

Whether patients were informed about the importance of oral health for their future EP was not associated with bleeding gums, their oral health and oral hygiene behaviour (*p* > 0.05, Table 2). Moreover, whether patients were informed on the importance of sufficient oral hygiene for their future EP was significantly associated with regular professional tooth cleaning; specifically, in the case of respective information, patients reported of professional tooth cleaning nearly twofold more often (59.8% vs. 35.6%, *p* = 0.01). Further associations were not observed (*p* > 0.05; Table 2).

**Table 2:** Associations between patient information about the importance of oral health or oral hygiene for EP and oral health issues (%)

	Information about oral health and EP		
	Yes	No	*p* value
*Bleeding gums*			
Yes	15.8	12.1	0.62
No	84.2	87.9	
*Dental visit prior to EP*			
Yes	28.1	13.6	0.05
No	71.9	86.4	
*Regular professional tooth cleaning*			
Yes	46.6	48.9	0.87
No	53.4	51.1	
*Toothbrush*			
Powered	39.0	37.5	0.86
Manual	61.0	62.5	
*Interdental cleaning*			
Yes	25.9	31.8	0.47
No	74.1	68.2	

	Information about oral hygiene and EP		
	Yes	No	*p* value
*Bleeding gums*			
Yes	14.4	11.1	0.79
No	85.6	88.9	
*Dental visit prior to EP*			
Yes	20.2	23.3	0.82
No	79.8	76.7	
*Regular professional tooth cleaning*			
Yes	59.8	35.6	0.01
No	40.2	64.4	
*Toothbrush*			
Powered	40.0	31.8	0.46
Manual	60.0	68.2	
*Interdental cleaning*			
Yes	34.0	25.0	0.33
No	66.0	75.0	

### Perspective of orthopaedic surgeons

In total, 221 orthopaedic clinics were included in the survey. Most of the clinics were Endo-Cert certified centres (66.1%), 9.0% were University Medical Centres; additionally, the majority of the EP centres were located in cities with more than 20,000 inhabitants (overall: 78.3%; 20,000–100,000: 37.1%; > 100,000: 41.2%). Among participating EP centres, nearly a third of the centres implant 100–249 EPs (33.9%), 250–500 EPs (30.8%) or more than 500 EPs (29.4%) annually.

Only a few centres had dental contact (14%), although the majority (92.8%) of the centres were familiar with causal relationships between oral health and EP infections. Less than half of the centres reported of referring their patients either verbally (48%) or in writing (43.9%) to the dentist. Moreover, approximately three-quarters of the centres stated of informing their patients on the necessity of antibiotic prophylaxis; however, the minority (7.2%) of the centres knew the appropriate drugs and dose for prophylaxis (2 g of amoxicillin or, in cases of allergies, 600 mg clindamycin, at 1 h prior to dental intervention) (Table 3).

**Table 3:** Consideration and management of oral health concerns by orthopaedic clinics in the context of EP implantation (% for/if yes)

Parameter	Patients before EP (n = 172)
Do you have a dental contact?	14.0
Do you take care on the oral health situation of your patients prior to EP?	71.0
Are you familiar with causal relationships between EP infections and oral health?	92.8
Do you refer your patients in writing to his or her dentist?	43.9
Do you refer your patients verbally to his or her dentist?	48.0
Do you have sufficient knowledge on oral health issues/dentistry?	23.5
Do you inform your patients on the necessity of an antibiotic prophylaxis for dental interventions after EP insertion?	76.0
The indication for an antibiotic prophylaxis should be confirmed by…	
Orthopaedic surgeon	69.2
Dentist	86.4
General practitioner	43.0
Antibiotics for antibiotic prophylaxis correct	7.2

**Fig. 1:**
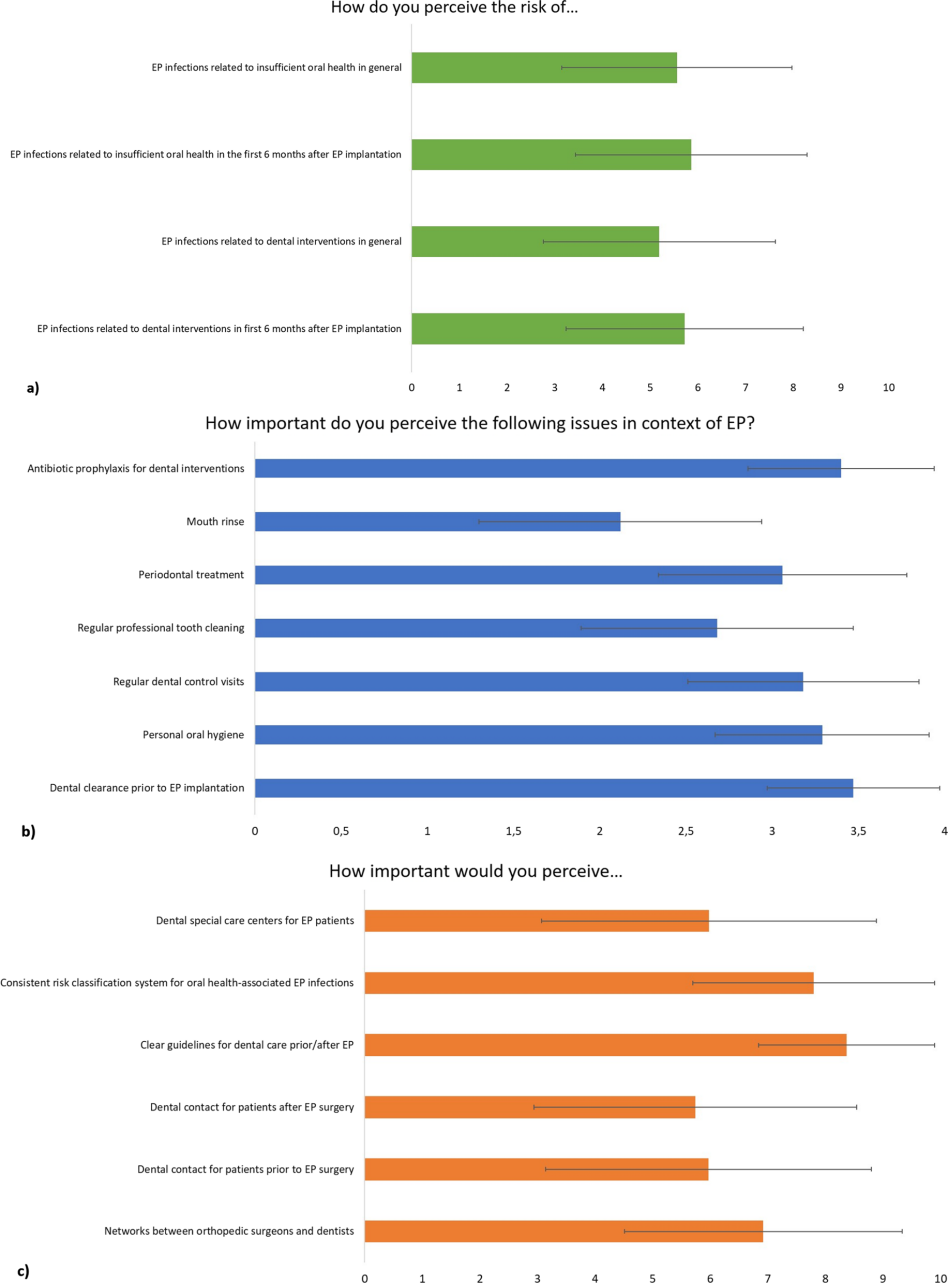
**a** Orthopaedic surgeons perceived risk for oral health-related infectious complications at EP. Ratings were made between very low = 0 and very high = 10. **b** Orthopaedic surgeons perceived importance of different oral health-related issues for the risk for infectious complications at EP. Ratings were made between unimportant = 0 and highly important = 4. **c** Orthopaedic surgeons perceived importance of different dental care-related issues with regard to EP. Ratings were made between unimportant = 0 and highly important = 10

Orthopaedic surgeons perceived the risk for oral health-related EP infections at an average of 5 to 6 points on a scale between 0 and 10 (Fig. 1a). As oral health-related factors with a potential influence on EP infections, orthopaedic surgeons attributed the highest importance to antibiotic prophylaxis, dental clearance prior to EP and personal oral hygiene (Fig. 1b). In terms of improving oral care in patients prior to EP, orthopaedic centres stated clear guidelines and a consistent risk-classification system as the most important issues (Fig. 1c).

### Associations between characteristics of the orthopaedic clinic and oral health concerns

The number of EPs (which centres reported on an annual basis) was not associated with their handling of oral health and dental care issues (*p* > 0.05; Table 4). University Medical Centres reported of more frequent dental contacts (*p* < 0.01). Further associations between the type of clinic and dental care-related issues were not observed (*p* > 0.05; Table 5). Moreover, centres in a city with 5000–19,999 (82.5%) and > 100,000 inhabitants (75.8%) reported more often of taking care of the oral health situation prior to EP than in cities with < 5000 (62.5%) and 20,000–100,000 inhabitants (61%, *p* = 0.04). Further associations between the number of inhabitants and dental care-related issues were not demonstrated (*p* > 0.05; Table 6).

**Table 4:** Associations between the number of patients annually treated with an EP and oral health-related issues (%)

	Number of EP annually				
	< 100	100–249	250–500	> 500	*p* value
*Do you have a dental contact?*					
Yes	23.1	8.0	17.6	15.4	0.26
No	76.9	92.0	82.4	84.6	
*Do you take care on the oral health situation of your patients prior to EP?*					
Yes	46.2	73.3	70.6	73.8	0.23
No	53.8	26.7	29.4	26.2	
*Antibiotics for antibiotic prophylaxis correct*					
Yes	0.0	8.0	7.4	7.7	0.78
No	100.0	92.0	92.6	92.3	
How important do you perceive a network between EP centres and dentists?	6.69 ± 2.18	6.73 ± 2.37	7.22 ± 2.11	6.88 ± 2.80	0.59
How important do you perceive a dental special care centre for patients prior/after EP?	7.00 ± 3.03	6.12 ± 2.75	5.34 ± 2.71	6.28 ± 3.18	0.08
*Do you have sufficient knowledge on oral health issues/dentistry?*					
Yes	7.7	25.3	23.5	24.6	0.57
No	92.3	74.7	76.5	75.4

**Table 5:** Associations between the characteristics of the clinic and oral health-related issues (%)

	Kind of clinic			
	University Medical Centre	Endo-Cert certified clinic	Others	*p* value
*Do you have a dental contact?*				
Yes	55.0	8.9	12.7	< 0.01
No	45.0	91.1	87.3	
*Do you take care on the oral health situation of your patients prior to EP?*				
Yes	70.0	69.2	76.4	0.60
No	30.0	30.8	23.6	
*Antibiotics for antibiotic prophylaxis correct*				
Yes	10.0	8.9	1.8	0.20
No	90.0	91.1	98.2	
How important do you perceive a network between EP centres and dentists?	7.45 ± 2.50	6.68 ± 2.43	7.36 ± 2.29	0.07
How important do you perceive a dental special care centre for patients prior/after EP?	6.95 ± 3.03	5.85 ± 2.82	5.96 ± 3.07	0.24
*Do you have sufficient knowledge on oral health issues/dentistry?*				
Yes	20.0	21.2	30.9	0.33
No	80.0	78.8	69.1

**Table 6:** Associations between the number of inhabitants in the city, where the clinic is located and oral health-related issues

	Inhabitants of the city				
	< 5000	5000–19,999	20,000–100,000	> 100,000	*p* value
*Do you have a dental contact?*					
Yes	0	17.5	9.8	17.6	0.27
No	100	82.5	90.2	82.4	
*Do you take care on the oral health situation of your patients prior to EP?*					
Yes	62.5	82.5	61	75.8	0.04
No	37.5	17.5	39.0	24.2	
*Antibiotics for antibiotic prophylaxis correct*					
Yes	0	2.5	7.3	9.9	0.41
No	100	97.5	92.7	90.1	
How important do you perceive a network between EP centres and dentists?	6.50 ± 3.12	7.10 ± 2.22	6.79 ± 2.43	7.00 ± 2.44	0.90
How important do you perceive a dental special care centre for patients prior/after EP?	4.88 ± 3.04	5.63 ± 2.97	5.90 ± 2.91	6.30 ± 2.87	0.42
*Do you have sufficient knowledge on oral health issues/dentistry?*					
Yes	12.5	25.0	24.4	23.1	0.89
No	87.5	75	75.6	76.9	

## Discussion

Only one-third of the patients prior to EP were informed about the potential relationship between EP and oral health. Regular professional tooth cleaning and interdental cleaning were comparably rare, thus indicating deficits in oral hygiene behaviour of the patients (especially in home care for interdental cleaning, as well as professional care involving regular professional tooth cleaning). However, information about oral hygiene and EP was associated with regular tooth cleaning. Although orthopaedic centres stated that they were familiar with the relationship between oral health and EP, dental collaboration and/or referral was not a common practice. Although several minor associations between the characteristics of the centre and their handling of dental health issues were confirmed, no clear difference was observed between different types of EP centres.

Based on the potential relationship between oral inflammation and EP infections, one may have expected that the oral health behaviour of patients prior to EP should be increased. However, the current study demonstrated that there is a lack of information on the oral hygiene behaviour of patients. In this regard, patients in this EP study cohort showed approximate equal rates of oral health behaviours compared to a representative German population of the Fifth German Oral Health Study (DMS V) at a similar age (65–74 years). Likewise, approximately one-third of participants used an electric toothbrush (EP: 35.5%, DMS V: 31.3%), as well as interdental cleaning (EP: 29.1% vs. DMS V: 28.6%). Although almost half (48.3%) of all of the patients prior to EP received regular professional tooth cleaning, this scenario only applied to 30% of the DMS V population [14].

Moreover, other patient groups exhibited deficits in oral hygiene and oral health behaviour; in particular, severely diseased patients showed insufficient oral behaviour, including patients with heart diseases, dialysis or organ transplantation [11, 15, 16]. Therefore, it has been concluded that patients with severe general diseases often exhibit a decreased perception of oral conditions (*response shift*) [17]. Patients prior to EP regularly suffer from pain in the respective joints [18]. Moreover, the impaired quality of life and pain may displace other health concerns, such as oral health. Therefore, patients foster neither their oral hygiene nor their prevention-oriented dental behaviour. This highlights the potential necessity of appropriate information and motivation of patients for oral health issues. When compared to previous findings of this working group, whereby one-third of patients prior to EP surgery had at least one potential oral focus [9], an improvement in oral behaviour would be highly recommended. As the results demonstrated, the minority of patients were informed about oral health and EP. Therefore, only information on the importance of oral hygiene for EP led to a higher utilisation of professional tooth cleaning (Table 4). Accordingly, more patient-oriented forms of information and sensibilisation may be needed, such as the application of visual metaphors or comprehensive information based on flyers, as has been previously performed [19]. Therefore, when regarding the average age of the patients, the EP study population also included elderly patients who may have been geriatric. In this context, the age-related increasing risk of (perioperative) inflammatory diseases due to the less efficiently working immune system (known as “immune senescence”) must be considered [20].

When considering the insufficient information and the oral behaviour of patients, the results of orthopaedic clinics are of considerable interest. Therefore, we observed that EP surgeons are aware of the importance of oral health and perceive its importance as being high (see Fig. 1); however, they refer only half of patients to a dentist, whereas the minority have dental contacts (Table 6). This scenario is similar to a small previous survey in 2011, although the previous study observed that only 3% of orthopaedic clinics had dental contacts [12]. Although evidence is limited, there appears to be a lack of cooperation between orthopaedic surgeons and dentists; this result has already been observed in the context of osteoporosis treatment and risk of osteonecrosis of the jaw [21], which is a far more evident and common topic than oral health-induced EP infections. Different views between dentists and other medical professionals, such as general physicians, have also been previously demonstrated [13]. Based on the separate education of dentists and physicians in Germany, divergent expectations and insufficient knowledge have appeared to result in a lack of collaboration [22, 23]. Therefore, this problem appears to require changes in education (undergraduate and postgraduate) to create awareness of the respective other fields. Another topic of the survey was the need for antibiotic prophylaxis for dental interventions in patients with EP. Seventy-six percent of EP centres stated of informing their patients on the necessity of antibiotic prophylaxis for dental interventions after EP insertion. This rate is considerably greater than that reported in a previous study (55%) [12]. However, current literature and international recommendations no longer recommend antibiotic prophylaxis for these patients [7, 8]. In contrast, recent clinical data suggest that dental rehabilitation and maintenance would be most appropriate to support EP health [7], which necessitates an improvement in the collaboration between dentists and orthopaedic surgeons. As Table 2 illustrates, only one-quarter of patients informed their dentist about the planned EP. Therefore, the main task in informing/educating patients appears to be focused on orthopaedic surgeons, who should refer the patients to a dentist prior to EP surgery.

Additionally, the current study examined the potential associations between the characteristics of the centre and their handling with oral health concerns. Although the number of inhabitants of the city in which the centre was located had an incongruent association with the centres’ care of oral health issues, another finding was of potential interest. Specifically, University Medical Centres had significantly more frequent dental contacts; this may be explained by the structure of university clinics, which often also have a dental faculty of dentistry, thus facilitating interdisciplinary collaboration. As shown in a previous study conducted within a university setting, this type of collaborative/cooperative partnership is a working but elaborative relationship [9]. Accordingly, in addition to improved interdisciplinary collaboration, easily applicable and practical solutions are needed to simplify the transfer of such concepts into broad practice.

This current questionnaire-based study applied two comprehensive surveys to both patients and orthopaedic surgeons, whereby a reasonable cohort of each group could be included. To the authors’ knowledge, these represent the largest surveys for both patients prior to EP and orthopaedic centres. The fact that centres throughout Germany were included ensured that the survey was quite representative. However, the study was restricted to Germany; therefore, the generalizability of the results to other countries with distinct health systems is limited. Additionally, patients were only surveyed in one centre. Moreover, the perspective of respective dentists is still missing. A similar questionnaire for the related dentists would have provided interesting information about the “other side” of the cooperation. Furthermore, the response rate of orthopaedic clinics was quite low but comparable with previous research [12]. This may have also limited the results, as the surgeons answering the questionnaire may have a certain interest or motivation in the topic of dental care. It can be presumed that a survey including all centres would have demonstrated an even worse situation.

In addition, EP patients are often seniors with specific geriatric problems. For this reason, it may be of interest to determine which centres provide an orthogeriatric specialty and consequently focus more on oral health than nonorthogeriatric centres. This point was not mentioned in the current survey of EP centres. Altogether, the current survey provides novel information and potential implications to improve the interdisciplinary care of patients prior to EP.

In summary, based on the current scientific evidence, a recommendation for dental examination and focal restoration (intervention) prior to EP can be suggested to prevent oral health statuses from becoming a potential risk for possible EP infections [7, 24]. Nevertheless, for practical implementation and a scientific outlook, interdisciplinary collaboration between orthopaedic clinicians and dentists is important and should be considered in future research. Further research efforts are required to prove causality between oral health and EP infections, including (1) evidence that dental visits and (as needed) focused rehabilitation (intervention) prior to EP reduces the number of infections compared to missing dental visits prior to EP, (2) the detection of oral pathogenic bacteria (both orally and on EP) at infected EP and (3) comparison with another patient group (under risk) that is particularly susceptible to oral-related EP infections [7, 24].

## Conclusion

Patients prior to EP exhibit deficits in oral hygiene behaviour and information on the potential relevance of oral health for their EP. Orthopaedic clinics show a lack of dental collaboration. Accordingly, improvements in interdisciplinary care, especially regarding practical concepts, appear to be necessary.

## Data Availability

The datasets that were used and/or analysed during the current study are available from the corresponding author on reasonable request. The data are not publicly available because of the pseudonymization and data protection guidelines according to the ethics approval.
